# Diversity of Phytochemical Content, Antioxidant Activity, and Fruit Morphometry of Three Mallow, *Malva* Species (Malvaceae)

**DOI:** 10.3390/plants14060930

**Published:** 2025-03-16

**Authors:** Faten Rhimi, Mokhtar Rejili, Mohamed Ali Benabderrahim, Hédia Hannachi

**Affiliations:** 1 Laboratory of Plant Productivity and Environmental Constraints (LR18ES04), Department of Biology, Faculty of Sciences of Tunis, University Tunis EL Manar, Tunis 2092, Tunisia; faten.rhimi@fst.utm.tn; 2 Department of Life Sciences, College of Sciences, Imam Mohammad Ibn Saud Islamic University (IMSIU), Riyadh 11623, Saudi Arabia; 3 Arid and Oases Cropping Laboratory LR16IRA02, Arid Lands Institute, Medenine 4119, Tunisia; mohamedali.benabderrahim@fst.utm.tn

**Keywords:** schizocarp, polyphenols, flavonoids, proteins, oil, starch, soluble sugar, image analysis, antioxidant activity

## Abstract

The Malvaceae family contains a variety of medicinal plants, including those of the mallow (*Malva)* genus. This study investigated the phytochemical composition, antioxidant activity, and morphometry of fruit from three *Malva* species (common mallow *M. sylvestris*, cheeseweed mallow *M. parviflora*, and Cornish mallow *M. multiflora*. The phytochemical analysis and antioxidant potentials were conducted on dry and powdered fruits. The morphometry and texture were performed using the software imagery tools. Results showed that the principal component analysis based on phytochemical content, antioxidant activity, and morphometric and texture traits showed a group structure according to species. The *M*. *multiflora* fruits were distinguished by high values of morphometric and texture traits, oil content (17.96%), total flavonoid content (1.70 mg RE/g DM), total antioxidant activity, and high FRAP value. The *M*. *parviflora* fruits were smaller than the *M*. *multiflora* and were rich in proteins (17.70%), starch (94.41 mg/g DW), and soluble sugar contents (57.74 mg/g DW). However, the *M*. *sylvestris* fruits have an intermediate position within the two other species and are rich in total polyphenols (2.34 mg GAE/g DM) and flavonoid contents (0.75 mg RE/g DM). The combined data, including phytochemical characterization, antioxidant activity, and morphometry, could be used as an alternative tool to molecular analysis to distinguish between *Malva* species and to select future application of *Malva* fruits based on their metabolite’s composition.

## 1. Introduction

The *Malva* genus, part of the Malvaceae family, includes common wild plants used in traditional medicine and shows potential activity against diseases related to the digestive system, respiratory disorders, and inflammation [1,2,3,4], and were also of culinary value [5]. *Malva* plants are most famous for their anti-inflammatory effect, which is attributed to bioactive compounds such as phenolics, which are also potent antioxidants in different organs (leaves, stem, flower, fruits) [1,2,3]. The use of medicinal plants as natural sources for drugs must be accompanied by a clear knowledge of species composition. Furthermore, their identification is crucial to avoid misuse such as incorrect dosage or the use of harmful or ineffective species [6].

The distinction of *Malva* species, including common mallow (*Malva sylvestris*), cheeseweed mallow (*Malva parviflora*), and Cornish mallow (*Malva multiflora)*, has traditionally relied on molecular markers such as DNA barcoding and microsatellites [7,8]. While molecular tools provide high accuracy, they can be costly, time-consuming, and require sophisticated laboratory equipment, which limits their widespread application, especially in regions with limited resources. In contrast, the use of phytochemical profiling and morphological traits offers a more accessible, cost-effective, and rapid approach to species differentiation. Phytochemical analysis, including the examination of secondary metabolites, has been shown to reveal unique chemical signatures that distinguish species with similar ecological niches.

Numerous studies have been published on the diversity of the *Malva* genus, focusing on morphological traits, chemical composition, and molecular markers [9,10,11,12]. The morphological characteristics of the stem, leaves, flowers, fruit, and seeds support the diversity within the *Malva* genus, with some species distinguishable by specific features [13]. The digital image was used as a tool to conduct morphological description and to extract morpho-colorimetric data on plant organs. The seed morphometric and colorimetric features characterizing size, shape, and textural seed parameters of 28 taxa belonging to the genera *Lavatera* L. and *Malva* L. were performed by using computer vision techniques [13]. The obtained results for *Malva* showed the high variation among taxa and supported the use of seed image analysis as a diagnostic for systematic purposes. Furthermore, the identification of *Malva* species/varieties and the evaluation of their genetic diversity were studied by flow cytometric genome size estimation and ISSR molecular markers on 12 selected accessions [14]. The Random Amplified Polymorphic DNA combined to morphometry traits were conducted to investigate the genetic variation among seven *Malva* species including *M*. *sylvestris* and *M. parviflora* [15]. These studies reported high genetic diversity, which clearly indicated that the *Malva* species can adapt to changing environments. Based on pollen morphometric characters, scanning electron microscopy and light microscopy were used to discriminate between two *Malva* species (*M. neglecta* and *M. parviflora*) [16]. The phytochemical and biological activities of extracts from leaves, flowers, and fruits were conducted on several species such as *M*. *sylvestris*, *M*. *parviflora* [1], *M*. *pseudolavatera* [4], and *M*. *aegyptiaca* [2]. Despite the valuable insights provided by morphological traits and texture characteristics, limited information exists on the integration of morphometry, texture, and phytochemical contents for *Malva* species characterization. Combining these approaches not only improves the accuracy of species distinction but also complements molecular data, offering a practical solution, particularly in cases where genetic resources are limited or inaccessible. Phytochemical studies hold significant potential for uncovering bioactive compounds that could lead to new medicinal applications of *Malva* species.

In this study, we focused on phytochemical, antioxidant, morphometric characters and texture traits of *Malva* fruits to highlight specific characters, their uses for fast and reliable species identification and their phytochemical potential. For this, fruits from three *Malva* species located in sympatry were used (*M*. *multiflora*, *M*. *parviflora* and *M*. *sylvestris*).

## 2. Results

### 2.1. Phytochemical Characteristics

**Figure 1:**
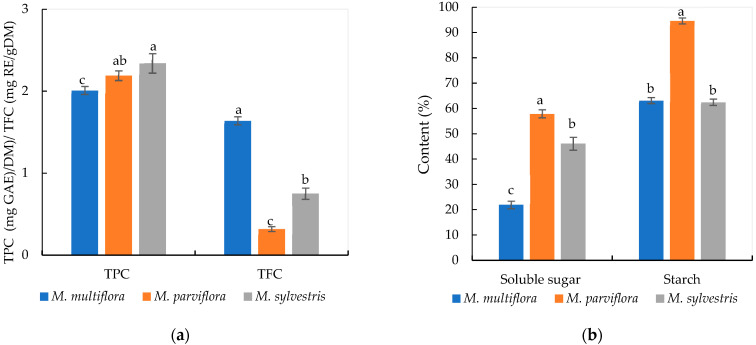
Polyphenols, flavonoids, soluble sugar, and starch contents of *Malva* fruits from the three studied species. (**a**): Total polyphenols content (TPC expressed as mg gallic acid equivalent per g dry matter) and total flavonoid content (TFC expressed as mg rutin equivalent per g dry matter); (**b**): soluble sugar and starch contents in mg per g dry matter. Different letters for the same content data showed significant difference at *p* < 0.05 using Duncan’s test.

The phytochemical contents showed that the *M*. *sylvestris* fruits were distinguished by having the highest TPC values of 2.34 mg GAE/g DM, followed by *M*. *parviflora* with 2.19 mg GAE/g DM, and *M*. *multiflora* (2.01 mg GAE/g DM). The *M. multiflora* fruits seemed to be rich in total flavonoid content (1.64 mg RE/g DM) compared to *M*. *sylvestris* (0.70 RE/g DM) and *M*. *parviflora* (0.30 RE/g DM) fruit extracts (Figure 1a). Results showed that the *M. parviflora* fruits have the highest soluble sugar (57.92 mg/g DM) and starch (94.67 mg/g DM) compared to *M*. *sylvestris* and *M*. *multiflora* (Figure 1b). Significant differences were noted within the TPC, TFC, soluble sugar, and starch according to the studied species.

**Figure 2:**
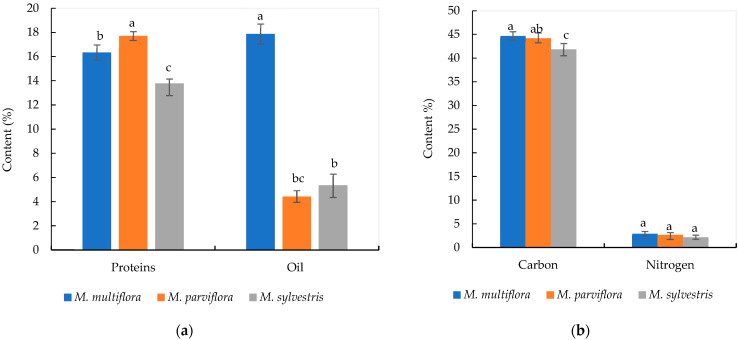
Protein, oil, carbon, and nitrogen contents of *Malva* fruits from the three studied species. (**a**): Proteins and oil contents in percentage; (**b**): carbon and nitrogen contents in percentage. Different letters for the same content data showed significant difference at *p* < 0.05 using Duncan’s test.

The *M. multiflora* fruits seemed to be rich in oil (17.87%), followed by *M*. *multiflora* with a percentage of 16.34, and *M*. *parviflora* having 13.78% oil (Figure 2a). The highest carbon value (44.72%) and nitrogen (2.96%) content were found in *M*. *multiflora* fruit (Figure 2b).

### 2.2. Antioxidant Activity

The antioxidant activity of Malva aqueous extracts was assessed using three tests: DPPH, FRAP, and Total Antioxidant Activity (TAA). The results indicated that all extracts exhibited antioxidant activity (Table 1). Among them, the fruit of M. multiflora showed the highest antioxidant activity in the TAA test, with a value of 22.01 mg AAE/g DM and a ferric reducing–antioxidant power (FRAP) of 58.811 as percentage of reductor power. In contrast, the fruit of M. parviflora demonstrated the strongest antioxidant activity in the DPPH test, with the lowest IC50 value of 0.01 mg/mL having an important potential for scavenging free radicals. M. sylvestris exhibited intermediate antioxidant potential in comparison to the other species.

### 2.3. Morphometric and Texture Characters of Malva Fruits

The morphometric (16 parameters) and texture (8 parameters) traits of *Malva* fruits from the three species studied were determined (Table S1). Analysis of variance revealed significant differences among species for most of the measured traits, except for three morphometric traits: Feret Angle, Angle, and the proportional relationship between the width and length of the fruit’s bounding rectangle, which were not significantly different (*p* > 0.05) (Table 2). Out of the 24 measured morphometric and texture traits, 9 traits exhibited high values that distinguished *M. multiflora*, including 8 morphometric traits and 1 texture trait. Notably, the area was significantly larger in *M. multiflora* (60.46 mm^2^), approximately 1.5 to 2 times greater than that of *M. parviflora* (30.00 mm^2^) and *M. sylvestris* (42.27 mm^2^) fruits. Regarding texture traits, the integrated density (the product of area and mean gray value) was also notably higher in *M. multiflora* fruits.

*M. parviflora* fruits were distinguished by the maximum gray value and skewness (the degree of asymmetry in the distribution of gray intensity values), which were more distinct compared to the other two species. In contrast, the mean and mode of the gray value texture traits effectively differentiated *M. sylvestris* fruits from the other species. Interestingly, the FeretX and FeretY values were highest and most similar in *M. parviflora* (FeretX = 329.90, FeretY = 413.67) and *M. sylvestris* (FeretX = 400.50, FeretY = 457.83), while *M. multiflora* fruits had significantly lower mean values of 119.40 and 121.57, respectively.

### 2.4. Multivariate Analysis

**Figure 3:**
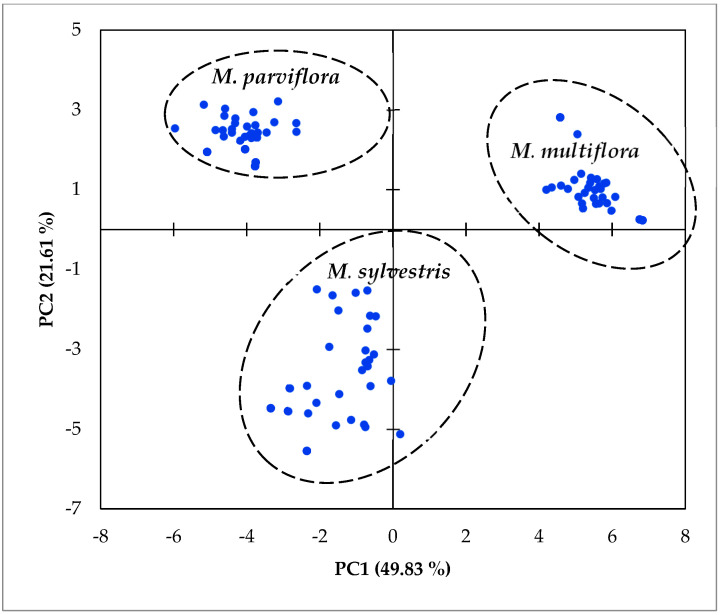
Principal component plot defined by the two first axes (PC1 and PC2) of principal component analysis applied on morphometry, texture, and phytochemical data of fruit from *Malva* species: *M*. *multiflora*, *M*. *parviflora*, and *M*. *sylvestris*.

The PCA is a statistical technique used to reduce the dimensionality of data while preserving as much variability as possible and is a valuable tool to improve the process of group structuration based on used data. The PCA was applied on phytochemical characteristics, antioxidant potential, morphometry, and texture. The first two axes of PCA explained 71.44% of total variation (Table S2). The first axis explained 49.83% of total variation and was correlated to 6 phytochemical data (TFC, sugar, starch, oil, TAA, and FRAP value), 11 morphometric parameters (area, perimeter, width, height, major, minor, circularity index, Feret, and MinFeret), and one texture (integrated density). However, the second PCA axis explained 21.62% of total variation and was correlated to 4 phytochemical parameters (TPC, C, N, proteins, and IC50) and 4 texture traits (median, mode, max gray value, and skew) (Table S1). The PCA plot, defined by the first two axes, clearly revealed three distinct groups corresponding to each species. The integration of phytochemical data with morphometric and texture traits not only enhances the structure of these species groups but also highlights their variation in terms of phytochemical contents having potential biological activities. This approach underscores the relationship between these traits and the species’ bioactivity, providing valuable insights into their medicinal potential (Figure 3, Figure S1).

## 3. Discussion

### 3.1. Phytochemical Data

With regards to phytochemical content and antioxidant potential, *Malva* fruits exhibited significant variation among species. Identifying which species have higher chemical concentrations can help pinpoint the most potent species for specific medicinal applications, such as anti-inflammatory, antimicrobial, or anticancer properties. The chemical variation found is linked to differences in the composition of both primary and secondary metabolites. The primary metabolites synthesized by plants are involved in the fundamental metabolic processes that are utilized by the plants for growth and development. The *M. parviflora* fruits were rich in soluble sugar and starch compared to the *M*. *multiflora* and *M*. *sylvestris*. In fact, some primary metabolites possess specific traits that are valuable for chemotaxonomic studies within the Malvaceae family (e.g., *Lavatera triloba*) [17], as well as in other families such as Bixaceae (e.g., *Bixa orellana*) [18] and both wild and cultivated olive trees (*Olea europaea)* [19]. Within the Malvaceae family, the chemotaxonomic studies on certain species have been conducted, with fatty acid content being considered a valuable taxonomic marker [20]. Da Silva et al. [21] have studied the fatty acid composition of oil seeds from seven species of the Malvaceae family as chemotaxonomic tools, and results supported the distinction between *Sida* and *Sidastrum*.

On the other hand, the secondary metabolites synthesized by plants are used for protection and defense mechanisms and they are very useful in chemotaxonomic classification including glycoside, alkaloid, volatile oil, flavonoids, polyphenols, and terpenoids [17]. The phenolic compounds constitute an essential group of secondary metabolites with a great diversity of structures [22] linked to the prevention of several diseases due to their pharmacological and biological activities [23].

Herein, we found that *M*. *sylvestris* fruits were rich in secondary metabolites compared to other species. This aligns with findings indicating the richness of this species in total polyphenols [1,24]. Beghdad et al. [25] reported that the leaf extracts of *M*. *sylvestris* have shown the highest amount of total phenolics (2.34 mg GAE/g DM) and total flavonoids (0.694 ± 0.017 mg RE/100 g) while the seed extracts presented the lowest values. However, Barros et al. [1] found that fruits from *M*. *sylvestris*, when compared to its leaves and flowers, revealed the lowest levels of nutraceuticals including phenols, flavonoids, and carotenoids. In comparison to the other species, the fruit extracts of *M. sylvestris* exhibited intermediate antioxidant potential with TAA assay (12.04 mg AAE/g DM) and FRAP (42.26%). In traditional medicine, *M*. *sylvestris* is widely used and it was investigated, with results showing an anti-inflammatory activity of fruit extracts [26] which supported the present results, showing that the aqueous extract from *M*. *sylvestris* fruits could be a source of bioactive polyphenols.

Regarding the *M*. *multiflora* fruits, they were distinguished by their high total flavonoid content and showcasing the highest antioxidant activity in the TAA test (22.01 mg AAE/g DM) and the FRAP (58.81%). This confirms the use of this wild plant as a “beqoula” dish (traditional North African salad) to meet the nutritional and therapeutic needs of the North African population, in particular [27]. Despite its traditional use as food and medicine, very limited information was found in the literature in regards to the phytochemical composition of this species.

Concerning *M. parviflora*, the fruits extracts exhibited the strongest antioxidant activity in the DPPH test (the lowest IC50 value of 0.01 mg/mL) with the amount of total phenolics (2.19 mg GAE/g DM) being slightly lower than *M. sylvestris*. The results of Naser et al. [28] demonstrate that *M. parviflora* leaf extracts have DPPH radical scavenging activity and might be used as a natural source for bioactive compounds. In contrast to *M. multiflora*, this species was widely studied for their chemical composition and antioxidant capacity. *M. parviflora* contains flavonoids, steroids, saponins, tannins, and terpenes in the water extract [28]. This confirms the uses of *M*. *parviflora* fruits in the Mediterranean diet and their characterization by their important biological activities [29]. Interestingly, *M. parviflora* mucilage extracted from its fruit and leaves can be used therapeutically for the management of inflammation, gastric ulcer, and coughing [29].

Divers’ methods were used to assess the antioxidant activity of plant extract [3,30,31,32]. It has been recommended to use different tests to evaluate the antioxidant potential of plant extract. For that, in this study, three different methods were used, including the ferric reducing–antioxidant power (FRAP), DPPH for scavenging radicals, and the TAA based on molybdenum reaction. Results showed that the fruit extract from the three studied *Malva* species exhibit antioxidant activity as shown previously on *M*. *neglecta* [33]. The use of *Malva* fruits in traditional food and medicine in different regions suggested their richness in compounds with antioxidant potential. It has been reported in different studies that the compounds having antioxidant potential in fruits and vegetables are the main factors explaining the efficacy of traditional foods in reducing the incidence of chronic diseases [34], which would explain by polyphenols with antioxidant capacity that could scavenge reactive chemical species. Some plant polyphenols are important components of human and animal diets and are safe for consumption [26,35], supporting the use of *Malva* fruits in foods. On other hand, the antioxidant compounds have a wide range of biological effects such as including antimicrobial, antiviral, anti-inflammatory activities [26,32].

In general, our results indicated that the extracts from the three fruit species exhibited antioxidant activity with significant variation. Accordingly, these species with antioxidant potential may be beneficial for preventive medicine or as dietary supplements to support overall health.

### 3.2. Morphometric and Texture

The morphometric and texture features based on imagery tools of seeds, stones, and fruits were used to study interspecific variation [15,17,36]. The imagery tool is useful to the morphometry study of plant organs and requires the use of geometric figures treated by software for decomposition of obtained shape variation into its symmetric and asymmetric components. The availability of digital image analysis software aids in the development of several morphometric indices [36]. The morphometry computer-assisted tools give the measurement of morphological traits of objects and are different from conventional Euclidean geometry. It was based on measurements using digital images containing some components of the image field as applied to our study on the 30 fruits per image. This method is based on threshold discrimination of objects by edge-finding algorithms. Each object in the image field was isolated by its true configuration and was converted to a binary image to conduct all measurements [17]. For this, in this study, the ImageJ 1xi free software accessed on 24 February 2024 (https://imagej.net/ij/) was used to treat digital images of *Malva* fruits from three species, converted into binary format, and to determine morphometric and texture features data. Thirty *Mava* fruits at three replicates (30 × 3) were subjected to morphometric and texture features measurement, followed by uni- and multivariate analyses. The collected data showed a significant specific effect (species variation) on measured parameters revealed through ANOVA and followed by Duncan’s test, confirming the significant morphometric differences among fruits from the three *Malva* species studied. Previously, morphometric studies have been investigated, and results showed the usefulness of these data in investigating the species and their varieties, as well as the environmental effect on seeds [36,37], stones [15], and tomato fruit shape [38]. While it has been reported that the *Malva* genus is morphologically diverse, some species are hardly distinguishable based on morphological traits [12]. The seed morphometry traits had been used to characterize *Malva* alliance [13]. Herein, the morphometry data combined with phytochemical characters could be used as efficient markers for species structure, as confirmed with the PCA analysis.

### 3.3. Multivariate Analysis

In this study, species characterization was carried out by considering the combination of all data, and results showed that the data integration of phytochemical, antioxidant potential, and morphometry allowed a clear species group’s structure. Accordingly, the total polyphenol and flavonoid contents could be used as phytochemical tools with morphometry and texture features to distinguish the *Malva* species. In this case, the data could be considered to valorize the *Malva* fruit in adequate nutritional and/or pharmaceuticals areas. The present results highlight that the *M*. *sylvestris* and *M*. *multiflora* were distinguished from *M*. *parviflora* in terms of secondary metabolites. The *M*. *parviflora* fruits were distinguished from the others by their nutritional reserves including protein, sugar, and starch contents. Thus, the phytochemical traits can complement morphological data in distinguishing *Malva* species and the adequate domain of valorization. This could be a promising tool for *Malva* species characterization. Both morphological characteristics and chemical composition are used to identify and classify medicinal plants, ensuring correct species identification for effective use in traditional medicine, particularly in ethnobotany and pharmacognosy. In future research, these findings will be applied on *Malva* species from different regions to further understand the environmental effects—through environment and species interaction—on morphometry and phytochemical content and to validate their uses in *Malva* species identification. The results of this study underline the potential of integrating phytochemical and morphological traits as consistent tools for species distinction in *Malva* species, offering an alternative to molecular markers, especially in settings with limited access to advanced genetic resources. Furthermore, our results highlighted the richness of these three *Malva* species in terms of their antioxidant potential. Moving forward, future studies could explore the broader applicability of these tools across other species within the Malvaceae family or other plant genera.

## 4. Materials and Methods

### 4.1. Malva Fruits Sampling

The fruit from three *Malva* species were harvested at maturity from the same region in northeastern Tunisia, Bizerte (37° 16′ N, 9° 52′ E). These species were in sympatry and share the same environmental conditions. Species identification is based on the Tunisian flora [39]. To conduct a randomized sampling, 10 plants were used for each species to harvest fruits (10 × 30), with a total of 300 fruits per species. The *Malva* fruit is called schizocarp, consisting of 10–12 glabrous wrinkled carpels transformed into achenes (mericarps) after double fertilization. Each achene contains one reniform seed.

The harvested *Malva* fruits were divided into two lots. One lot was used for morphometric analysis; the second one was used to proceed with the phytochemical determination. For that, fruits were dried in an oven at 40 °C until weight stabilization, then ground into a powder using an electric grinder, and stored in dark glass bottles at 4 °C until analysis.

### 4.2. Preparation of the Fruit Extract

The aqueous extraction by maceration was conducted using 1 g of dry, homogenized powdered fruits immersed in 10 mL of distilled water. The mixture was kept under magnetic stirring, incubated for 24 h in the dark and at 25 °C. Finally, the solutions were filtered using Wattman filter paper and the obtained extract was labelled and stored at 4 °C until use [31]. All chemicals and reagents were purchased from Sigma-Aldrich Co. (St. Louis, MO, USA) to conduct chemical analysis.

### 4.3. Determination of Total Polyphenols Content

The total polyphenols content (TPC) was estimated using the Folin–Ciocalteu method with modifications. A quantity of 63 μL of extract sample was mixed with 63 μL of the Folin–Ciocalteu reagent in a 96-well plate. After 5 min, 625 μL of 7.5% sodium carbonate (Na_2_CO_3_) was added. The solution was incubated in the dark at room temperature for 90 min and the absorbance reading was carried out at 760 nm using the UV/VIS microplate spectrophotometer. The control was prepared under the same conditions, with the extract being replaced with distilled water. The TPC was calculated from the calibration curve using gallic acid as the standard, and the results were expressed in milligram gallic acid equivalent per gram of dry matter (mg GAE/g DM) [31].

### 4.4. Determination of Total Flavonoid Content

The total flavonoid content (TFC) was determined by the aluminum trichloride method. A quantity of 125 μL of each extract was mixed with 38 μL of a sodium nitrite NaNO_2_ solution in a 96-well plate. After 6 min, 75 μL of 10% freshly prepared AlCl3 solution was added. Then, 250 μL of sodium hydroxide solution (NaOH) and 762 μL of distilled water were added to the mixture. After 5 min, the absorbance was measured at 510 nm using the microplate UV/VIS spectrophotometer. The TFC was expressed in milligram rutin equivalent per gram of dry matter (mg RE/g DM) based on a calibration curve using rutin as standard [31].

### 4.5. Determination of Protein Content by LECO Analyzer

Protein content was determined by elemental microanalysis as % nitrogen content x 6.25 using a LECO CHNS-932 analyzer (St. Joseph, MI, USA). This analysis was performed on 1 g sample of dry matter, packed in aluminum foil without any solvent and placed in specific wells of the apparatus. The display indicates the protein content (%) [40].

### 4.6. Determination of Oil Content

The oil content of Malva fruits was determined by the Soxhlet method. For this, 10 g of powdered fruits were placed into the thimble of the Soxhlet chamber. A volume of 200 mL of petroleum ether was used as extraction solvent in a round bottom flask and assembled for Soxhlet extractor. After complete continuous extraction for 6 h at 65 °C, the petroleum ether was removed by rotary vacuum evaporator [19]. The oil content was expressed as percentage using the following formula: Oil content (%) = [(W1 − W2)/W] × 100, where W1 = Initial sample weight placed in the thimble; W2 = Final sample weight obtained after drying by rotary vacuum evaporator.

### 4.7. Total Soluble Sugar and Starch Contents

The extraction was carried out using 10 mg of DM dissolved in 1 mL of 80% ethanol. The mixture was placed in a water bath at 70 °C for 30 min. Afterwards, the solution was cooled on ice, followed by centrifugation for 15 min at 9000 rpm at 4 °C. The obtained supernatant was used for sugar quantification and the pellet for starch quantification. A volume of 50 µL of supernatant was mixed with 2.5 mL of anthrone and 1200 µL of 80% ethanol. The solution was placed at 100 °C in a water bath for 10 min, then cooled on ice. The absorbance was measured at 640 nm. For starch quantification, 1 mL of 80% ethanol was added to the pellet, followed by centrifugation for 15 min at 9000 rpm at 4 °C. After filtration, 1 mL of the obtained supernatant was mixed with 30 µL of 35% perchloric acid and kept for 30 min in ice. Next, 2.5 mL of anthrone was added to 1200 µL of 80% ethanol and 100 µL of the starch extract. The obtained solution was stirred, then placed in a 100 °C water bath for 10 min. Subsequently, the solution was cooled, and the absorbance was measured at 640 nm [41]. Calibration was prepared using a glucose solution as a standard, and a control was prepared under the same experimental conditions using 80% ethanol. The sugar and the starch contents were expressed as mg per gram of dry matter (mg/g DM).

### 4.8. Antioxidant Activity

#### 4.8.1. Total Antioxidant Capacity

This assay is based on the reduction of Mo(VI) to Mo(V) by the extract and, subsequently, the formation of a green phosphate/Mo(V) complex at acid pH [42,43]. An aliquot of 0.1 mL of each extract was combined to 1 mL of reagent solution (0.6 M sulfuric acid, 28 mM sodium phosphate, and 4 mM ammonium molybdate). The tubes were incubated at 95 °C for 90 min. After that, the mixture was cooled at room temperature and the absorbance of each solution was measured at 695 nm against a blank. The ascorbic acid was used as standard, and the total antioxidant capacity (TAC) was expressed as mg ascorbic acid equivalents per gram dry matter (mg AAE/g DM).

#### 4.8.2. Radical Scavenging Ability

The radical scavenging activity of the extracts was measured using the DPPH (1,1-diphenyl-2-picrylhydrazyl) assay as described [32]. For this, 1 mL of the extracts at various concentrations were mixed with 0.25 mL of a DPPH-methanolic solution (0.2 mM) and were allowed to react in the dark for 30 min. Then, the absorbance of the resulting solution was measured at 517 nm. A control using distilled water until the extract was prepared under the same conditions. The inhibition of the percentage of the extracts was calculated at each concentration to determine IC50 (g/mL).

#### 4.8.3. Ferric Reducing–Antioxidant Power (FRAP)

The ferric reducing–antioxidant power (FRAP) measures the ability of the tested extracts to reduce ferric iron (Fe^3+^) present in the K_3_Fe(CN)6 complex to ferrous iron (Fe^2+^). A volume of 100 µL of different concentrations of each extract or ascorbic acid as a reference were added to 0.15 mL of phosphate buffer (0.2 M, pH 6.6) and 0.25 mL of ferricyanide (1%) and incubated at 50 °C for 20 min. After incubation, 0.35 mL of 10% tetrachloroacetic acid (TCA) was added and centrifuged at 3000× *g* for 10 min. Then, 0.25 mL of the obtained supernatant was added to 0.5 mL of 1% FeCl_3_ and 4 mL of H_2_O. The absorbance was immediately measured at 700 nm against a control prepared under the same conditions. The results were expressed as FRAP-reducing capacity (%) as follows: (Aa − Ab)/Ab × 100 with Aa the extract absorbance, Ab ascorbic acid absorbance [35].

### 4.9. Morphometric and Texture Analysis

**Figure 4:**
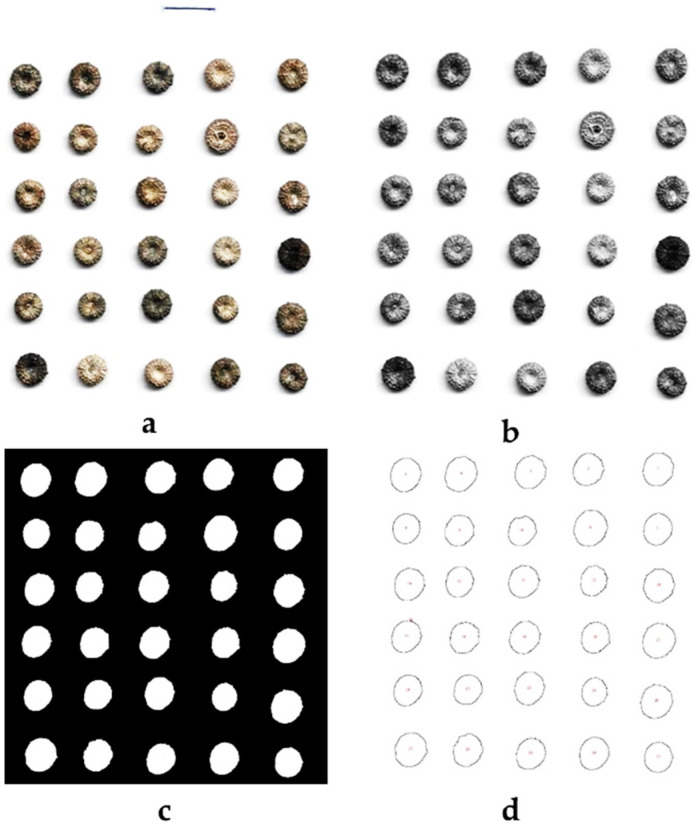
A summary of the method used to determine the morphometry of *M*. *parviflora* fruit. (**a**): Fruits image obtained by scanning; (**b**): basic image; (**c**): binary image; (**d**): profiles projections of fruits. The horizontal line shows the scale which corresponds to 1 cm.

**Figure 5:**
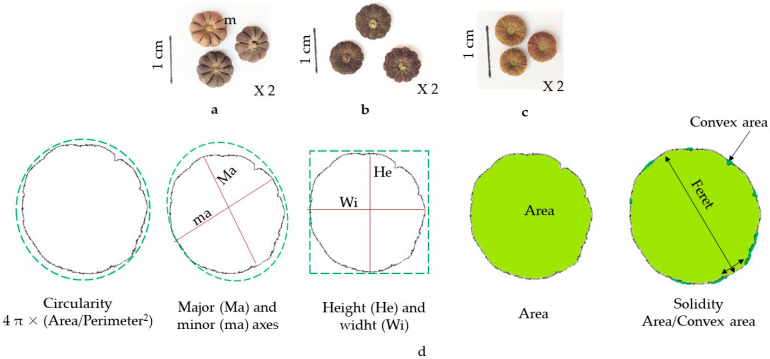
Phenotype of *Malva* fruits of the studied three species: *M*. *multiflora* (**a**), *M*. *sylvestris* (**b**), and *M*. *Parviflora* (**c**), as well as schematic morphometry parameters applied on *Malva* fruits (**d**) (m: mericarp).

The morphometric and texture measures were determined using the free software ImageJ (https://imagej.net/ij/) download the 24 February 2024. For each species, 30 fruits were randomly used three times to obtain digital images using HP Scanner, accompanied by a known scale which was used for scale-setting, and analysis started by the software ImageJ. Then, the images were converted into basic format, followed by binary making (Figure 4). The fruit projections obtained were analyzed as indicated previously [15,16,36]. The computer-assisted image analysis using the software ImageJ was utilized to determine 16 morphometric traits including those of size (area, length, width, and perimeter) and of shape (roundness and circularity index) and 8 texture features traits. Table 1 and Figure 5 showed the studied parameters as described previously [44].

### 4.10. Statistical Analyses

All data were expressed as mean ± standard deviations and were subjected to one-way analysis of variance analysis (ANOVA), followed by Duncan’s range multiple tests. In addition, the principal component analysis (PCA) was conducted on morphometry, texture features, and phytochemical data. Statistical analyses were performed using XLSTAT 2022 1.2. software accessed on 5 march 2024 (www.XlSTAT.com).

## 5. Conclusions

In this study, phytochemical content and morphometric traits of fruits from three *Malva* species were analyzed. *M. multiflora* was distinguished by its high values in morphometric traits, texture, oil content, total flavonoid content, and total antioxidant activity. The *M. parviflora* fruits were smaller and had higher levels of proteins, starch, and soluble sugars. *M. sylvestris* fruits exhibited intermediate characteristics between the other two species and were notably rich in total polyphenol content. These findings suggest that *Malva* fruits hold significant potential for use in natural medicine, with each species offering a specific profile of phytochemical compounds. The high flavonoid content and antioxidant activity in *M. multiflora*, for example, could support its use in formulations aimed at combating oxidative stress-related conditions. The elevated protein and starch levels in *M. parviflora* may be valuable in developing nutrient-rich supplements or functional foods. Meanwhile, the polyphenol-rich *M. sylvestris* may be explored for its potential anti-inflammatory or antimicrobial properties. The combined data, including phytochemical traits, morphometric, and texture analysis, can guide the selection of *Malva* species for future natural medicine applications based on their richness in primary or secondary metabolites and antioxidant activity. This approach not only enhances the medicinal potential of *Malva* but also offers a framework for sustainable harvesting and utilization of these species.

Finally, future work could involve refining phytochemical markers through advanced techniques, such as mass spectrometry or high-performance liquid chromatography, to enhance resolution and increase the number of chemical profiles used for species differentiation. This would pave the way for more targeted and effective applications of *Malva* in natural health products.

## Figures and Tables

**Table 1:** Antioxidant activity of fruit extract from three Malva species.

	TAA (mg AAE/g DM)	FRAP (% RP)	DPPH (IC50) (mg/mL)
*M*. *mulriflora*	22.01 ± 0.86 ^a^	58.81 ± 0.006 ^a^	0.20 ± 0.00 ^c^
*M*. *parviflora*	11.47 ± 2.02 ^b^	42.26 ± 0.022 ^c^	0.01 ± 0.00 ^a^
*M*. *sylvestris*	12.04 ± 0.94 ^b^	49.21 ± 0.047 ^b^	0.02 ± 0.00 ^b^

TAA: total antioxidant activity; AAE: ascorbic acid equivalent; DM: dry matter; RP: reductor power. Different letters in the same column showed significant difference at *p* < 0.05 using Duncan’s test.

**Table 2:** Parameters related to size, shape, and texture features of *M*. *multiflora*, *M*. *parviflora*, and *M*. *sylvestris* fruits.

	*M*. *multiflora*	*M*. *parviflora*	*M*. *sylvestris*
**Morphometry parameters**			
Area	60.46 ± 5.91 ^a^	30.00 ±3.37 ^c^	42.27 ± 5.17 ^b^
Perimeter	28.69 ± 1.48 ^a^	21.15 ± 1.16 ^c^	24.87 ± 1.47 ^b^
Width	8.74 ± 0.39 ^a^	6.14 ± 0.40 ^c^	7.28 ± 0.50 ^b^
Height	9.14 ± 0.34 ^a^	6.40 ±0.35 ^c^	7.55 ± 0.48 ^b^
AR	1.10 ± 0.03 ^a^	1.13 ± 0.02 ^a^	1.12 ± 0.08 ^a^
Major	9.26 ± 0.42 a	6.54 ± 0.36 ^a^	7.72 ± 0.49 ^b^
Minor	8.38 ± 0.30 ^a^	5.81 ± 0.35 ^c^	6.93 ± 0.45 ^b^
Angle	61.31 ± 21.04 ^a^	62.00 ± 7.95 ^a^	59.67 ± 10.28 ^a^
Circularity	0.92 ± 0.02 a	0.84 ± 0.04 ^c^	0.87 ± 0.04 ^b^
Feret	9.82 ± 0.43 ^a^	6.73 ± 0.39 ^c^	7.99 ± 0.50 ^b^
FeretX coordinates	119.40 ± 82.95 ^b^	329.90 ± 214.08 ^a^	400.50 ± 279.55 ^a^
FeretY coordinates	121.57 ± 63.05 ^b^	413.67 ± 216.76 ^a^	457.83 ± 233.90 ^a^
Feret Angle	58.20 ± 23.13 ^a^	59.78 ± 15.11 ^a^	57.50 ± 23.24 ^a^
MinFeret	8.65 ± 0.35 ^a^	5.94 ±0.33 ^c^	7.06 ± 0.43 ^b^
Roundness index	0.91 ± 0.03 ^a^	0.87 ± 0.07 ^b^	0.89 ± 0.06 ^ab^
Solidity	0.94 ± 0.01 ^b^	0.96 ± 0.01 ^a^	0.96 ± 0.03 ^a^
**Texture features**			
Mean of gray values	87.17 ± 5.93 ^b^	89.34 ± 5.01 ^b^	115.38 ±19.15 ^a^
Standard deviation	29.02 ± 3.79 ^a^	24.42 ± 2.93 ^b^	24.68 ±7.92 ^b^
Integrated Density	5493.17 ± 559.15 ^a^	2949.85 ± 311.74 ^c^	4677.07 ± 722.61 ^b^
Mode of gray value	87.10 ± 9.27 ^b^	88.77 ± 8.73 ^b^	124.64 ± 7.37 ^a^
Minimum of gray value	30.47 ± 9.65 ^a^	20.08 ± 10.11 ^b^	25.42 ± 11.71 ^a^
Maximum of gray value	165.67 ± 0.71 ^b^	170.00 ± 0.00 ^a^	165.03 ± 0.19 ^c^
Skewness	0.67 ± 0.37 ^b^	1.17 ± 0.51 ^a^	−0.73 ±0.82 ^c^
Kurtosis	0.39 ± 0.68 ^b^	1.62 ± 0.97 ^a^	2.33 ± 0.49 ^a^

Different letters in the same line showed significant difference at *p* < 0.05 using Duncan’s test

## Data Availability

All data generated or analyzed during this study are available within the article and the Supplementary File.

## Supplementary Materials

The following supporting information can be downloaded at: https://www.mdpi.com/article/10.3390/plants14060930/s1, Figure S1. Principal component plot defined by the two first axes (PC1 and PC2) of principal component analysis based on morphometry, texture traits, chemical content, and antioxidant activity of fruit of Malva species: *M*. *multiflora* (Mm), *M*. *parviflora* (Mp), and *M*. *sylvestris* (Ms) with label of points; Table S1. Morphometry and grayscale texture traits from binary imaging applied on the fruits of the three *Malva* species; Table S2. Principal component analyses applied morphometry, texture, and phytochemical data of *Malva* fruits.
