# Supplementary material for: Diversity of Phytochemical Content, Antioxidant Activity, and Fruit Morphometry of Three Mallow, Malva Species (Malvaceae)
Source: Plants (Basel). 2025 Mar 16;14(6):930. doi: 10.3390/plants14060930 (PMC11945817; doi:10.3390/plants14060930)
Supplement: Supplementary file 1 [file plants-14-00930-s001.zip › plants-3460970-supplementary.pdf]

Table S1. Morphometry and grayscale texture traits from binary image applied on the fruits of the three *Malva* species.

Parameters	Description
**Morphometry**	
Area	- The fruit surface calculated from the limit defined by the perimeter (mm^2).
Perimeter	- The length of the fruit contour (mm).
Bounding Rectangle	- Width and height measurements defined by the smallest rectangle enclosing the selected fruit image (mm).
	- Proportional relationship between width and length.
Surrounding ellipse	- Major and minor axes of the ellipse surrounding the selected fruit image (mm);
	- The angle between the major axis and a line parallel to the x-axis of the fruit image (0-180 degrees).
Circularity index	- This descriptor is obtained by $(4\pi * \text{Area})/\text{Perimeter}^2$ [12].
Feret's diameters	- Feret: the longest traceable diameter with two points of the fruit outline as endpoints (mm).
	- MinFeret diameter: the shortest distance between two points along the fruit selection boundary (mm) and perpendicular to the Feret.
	- FeretAngle (0-180 degrees): the angle between the Feret's diameter and a line parallel to the x-axis of the image.
	- FeretX and FeretY : the coordinates of the Feret's diameter.
Roundness index	- Fruit roundness index [14] is defined as $(4 * \text{Area})/ (\pi\ [\text{major axis}]^2)$.
Solidity	- Shape descriptor that describes the overall concavity of a fruit and defined as area/ convex area. The convex area is a delimiter of the original shape of the image, tightly enveloping the area, without indentations.
**Texture features from grayscale image**	
Standard deviation	- Intensity standard deviation as contrast measure.
Integrated Density	- The product of area and mean gray value.
Mean	- Average gray value within the selection.
Mode	- Mode of the gray values.
Min gray values	- Minimum gray value within the selection.
Max gray values	- Maximum gray values within the selection.
Skewness	- Asymmetry degree of intensity values distribution (grey levels).
Kurtosis	- Peakness degree of intensity values distribution.

Table S2. Principal component analyses applied on morphometry, texture and phytochemical data of *Malva* fruits

	PCA2-F1	PCA2-F2
Eigenvalue	14.51	7.30
Variability (%)	50.03	25.18
Cumulated variability (%)	50.03	75.20
Morphometry parameters		
Area	**0.930**	0.019
Perimeter	**0.878**	0.053
Width	**0.912**	0.033
height	**0.939**	0.019
Major axis	**0.925**	0.026
minor axes	**0.937**	0.029
Circularity index	0.514	0.004
Feret	**0.944**	0.019
FeretX coordinates	0.193	0.042
FeretY coordinates	0.348	0.093
FeretAngle	0.002	0.015
MinFeret diameter	**0.951**	0.021
Roundness index	0.050	0.016
Solidity	0.254	0.046
Texture parameters		
Standard deviation	0.177	0.069
Integrated Density	**0.693**	0.194
Mean of gray value	0.051	**0662**
Mode of gray values	0.054	**0.770**
Min gray value	0.097	0.024
Max gray value	0.378	**0.538**
Skewness	0.001	**0.760**
Kurtosis	0.122	0.142
Phytochemical data		
Total polyphenols content	0.348	**0.490**
Total flavonoids content	**0.945**	0.013
Soluble sugars	**0.948**	0.002
Starch content	**0.490**	0.455
C %	0.113	**0.633**
N %	0.239	**0.618**
%Proteins	0.011	**0.873**
%Oil	**0.894**	0.043
TAA	**0.885**	0.047
DPPH	0.283	**0.348**
FRAP	**0.937**	0.017

PCA: Principal component analysis; F1: first axis of principal component analysis F2: second axis of principal component analysis; morphometry parameters codes were explained in Tbale1. TPC: total polyphenols content; TFC: total flavonoids content; SSug: soluble sugar; star: starch content; Prot: protein. The bold values indicate the high correlation coefficient; AR: Proportional relationship between width and length of bounding rectangle

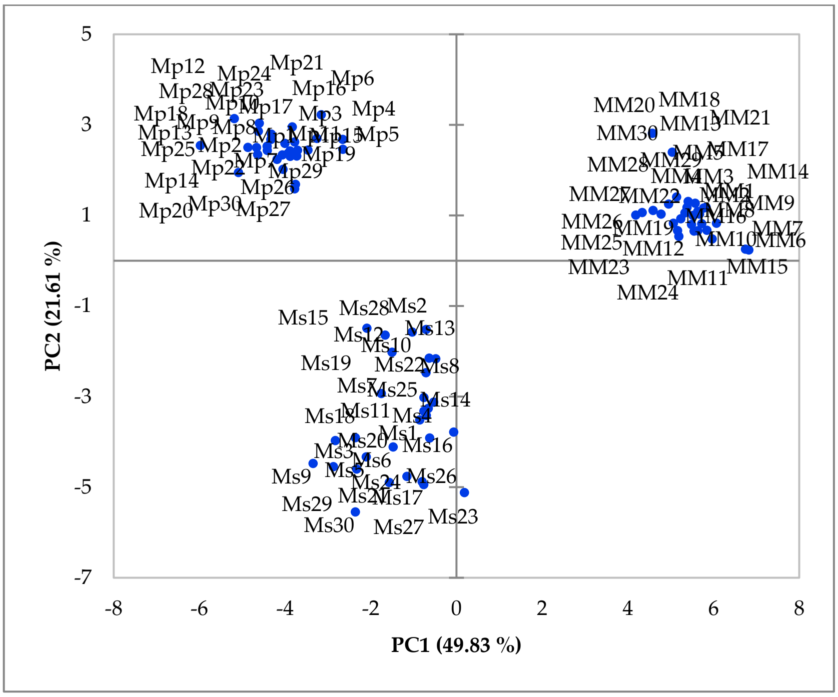

Figure S1. Principal component plot defined by the two first axes (PC1 and PC2) of principal component analysis applied on morphometry, texture and phytochemical data of *Malva* species: *M. multiflora* (Mm), *M. parviflora* (Mp) and *M. sylvestris* (Ms) with labelled points
